# Deep learning-based segmentation of acute pulmonary embolism in cardiac CT images

**DOI:** 10.1007/s11548-025-03503-0

**Published:** 2025-09-25

**Authors:** Ehsan Amini, Georg Hille, Janine Hürtgen, Alexey Surov, Sylvia Saalfeld

**Affiliations:** 1 Department of Medical Informatics and Statistics, University Hospital Schleswig-Holstein, Campus Kiel, Arnold-Heller-Str. 3, 24118 Kiel, Schleswig-Holstein Germany; 2 Institute for Medical Engineering, Otto-von-Guericke-University Magdeburg, Universitätsplatz 2, 39106 Magdeburg, Sachsen-Anhalt Germany; 3 Department of Radiology, Neuroradiology and Nuclear Medicine, Johannes Wesling University Hospital, Ruhr University Bochum, Hans-Nolte-Str. 1, 32429 Minden, North Rhine-Westphalia Germany

**Keywords:** Acute pulmonary embolism, Deep learning, CNN, Transformer, CTPA

## Abstract

**Purpose:**

Acute pulmonary embolism (APE) is a common pulmonary condition that, in severe cases, can progress to right ventricular hypertrophy and failure, making it a critical health concern surpassed in severity only by myocardial infarction and sudden death. CT pulmonary angiogram (CTPA) is a standard diagnostic tool for detecting APE. However, for treatment planning and prognosis of patient outcome, an accurate assessment of individual APEs is required.

**Methods:**

Within this study, we compiled and prepared a dataset of 200 CTPA image volumes of patients with APE. We then adapted two state-of-the-art neural networks; the nnU-Net and the transformer-based VT-UNet in order to provide fully automatic APE segmentations.

**Results:**

The nnU-Net demonstrated robust performance, achieving an average Dice similarity coefficient (DSC) of 88.25 ± 10.19% and an average 95th percentile Hausdorff distance (HD95) of 10.57 ± 34.56 mm across the validation sets in a five-fold cross-validation framework. In comparison, the VT-UNet was achieving on par accuracies with an average DSC of 87.90 ± 10.94% and a mean HD95 of 10.77 ± 34.19 mm.

**Conclusions:**

We applied two state-of-the-art networks for automatic APE segmentation to our compiled CTPA dataset and achieved superior experimental results compared to the current state of the art. In clinical routine, accurate APE segmentations can be used for enhanced patient prognosis and treatment planning.

## Introduction

Acute pulmonary embolism (APE) resulting from clots, fat, or other obstructive substances that block the pulmonary artery and disrupting the blood supply to lung tissues is the third most common cardiovascular condition [1]. Its symptomatic presentation ranges between asymptomatic and highly mortal, with mortality rates up to $30\%$  [2]. The most impactful prevention of severe consequences due to APE lies in the immediate and correct diagnosis with subsequent treatment.

For APE diagnosis, computer tomographic pulmonary angiography (CTPA) is considered as gold standard and derived parameters like right ventricle enlargement or epicardial adipose tissue are related to the 30-day mortality in APE patients [3, 4]. In addition, accurate delineation of the location, volume, and morphology of APE is crucial for risk stratification for APE patients to establish appropriate treatment and management. For example, a thrombolytic treatment and close surveillance in intensive care settings is advised for high risk patients [5]. In contrast, low-risk patients may be treated with anticoagulation without intensive care [6].

APE segmentation is a complex and demanding task due to factors such as the small size of emboli, variability in their appearance, and the need for high precision in delineation to avoid misdiagnosis [7]. Manual segmentation is time-consuming, labor-intensive, and highly subjective, relying heavily on the expertise of radiologists [7]. Especially when considering that each patient can have hundreds of CTPA image slices where emboli are covering more than 200 slices and comprise disconnected areas.

As a result, recent approaches aim at a computer-aided detection for the accurate detection and diagnosis of APE in order to reduce the CTPA reading time. Deep learning-based medical image analysis can cope with these strong variations with objectives like localizing, classifying and segmenting thrombi in CTPA imaging. There are various works utilizing rather traditional convolutional neural network (CNNs) architectures for automatic diagnosis and classification of APE in CTPA images. Some studies only classify whether a specific CTPA scan could be labeled as APE positive or negative, which simplifies the task to only assign categorical labels [8, 9]. However, such categorical assessments miss out on more quantitative information, e.g., precise position, extent, and vessel obstruction, which play an important role in APE patient risk stratification. Some of those limitations could be overcome by automated detection approaches of individual APE candidates [10–13]. In contrast, only few studies focused on semantic segmentation of APE, what would provide most comprehensive image-based analysis capabilities. For instance, Liu et al. [14] proposed a U-shaped network architecture with coordinate attention and pyramid pooling to segment APEs located in the major pulmonary arteries in 2D CTPA images. Pu et al. [15] proposed a two-step approach, consisting of an adapted UNet for pulmonary artery segmentation and a subsequent APE determination based on adaptive thresholding of the contrast between APE candidate and surrounding artery tissue. For training and validation purposes, volumetric image patches of CTPA scans were used. More recently, Chen et al. [16] presented a network architecture including Swin transformer blocks and multi-fusion dense skip connections, which was applied to 2D CTPA images of two publicly available datasets.

In contrast to the above mentioned, this study analyzes the potential of two state-of-the-art network variants for APE segmentation in whole 3D CTPA images.

## Materials and methods

### Imaging data

The presented work is part of a retrospective study that was approved by the institutional review board (Nr. 145/21, Ethics Committee, Otto-von-Guericke University of Magdeburg, Magdeburg, Germany). From the original study (2015–2021), comprising 508 datasets [4], a subset of 200 volumetric CTPA scans was randomly chosen for manual segmentation as ground truth.

The manual segmentation was conducted within the freely available MeVisLab 3.1 framework (Fraunhofer MEVIS and MeVis MedicalSolutions AG, Germany; https://www.mevislab.de), utilizing the CSO functionalities in cooperation with experienced radiologists.

Manual segmentation of APE was time-intensive and took approximately 1 h per patient, which was applied to approx. 80 datasets. After an initial training phase, we created predictions for unseen datasets to increase our sample size to 200 datasets. The predictions were manually analyzed and corrected with the same MeVisLab framework in close collaboration with two experienced radiologists. With such an iterative strategy, the required time effort for manual annotations was reduced to approximately 15 to 20 min per patient.

**Fig. 1:**
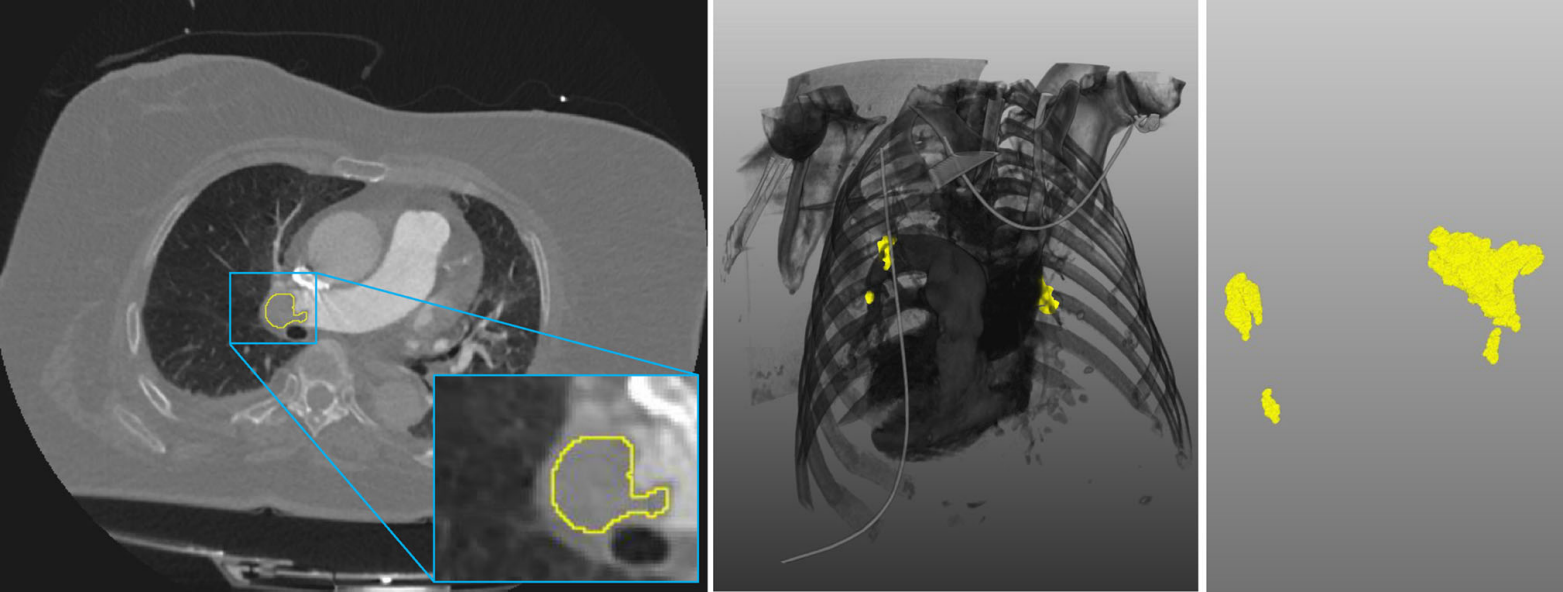
Illustration of an exemplary patient case with the ground truth APE contour (yellow). In the center, a direct volume rendering of the patient dataset in combination with the APE volume (yellow) is shown. On the right, only the 3D APE volume is depicted

The obtained dataset consists of 3D images from 200 patients with a voxel size (mm) of $0.782 \times 0.782 \times 1.0$, slice spacing of 1 mm, and matrix dimensions of $512 \times 512 \times 300-500$. An exemplary patient case is shown in Fig. 1.

**Table 1:** Performance metrics of the nnU-Net and VT-UNet adapted to our dataset and across five folds

Fold	nnU-Net		VT-UNet	
	DSC (%)	HD95 (mm)	DSC (%)	HD95 (mm)
Fold 0	87.73 ± 13.19	7.68 ± 22.86	86.94 ± 12.79	8.93 ± 23.03
Fold 1	89.04 ± 8.37	5.59 ± 11.32	89.06 ± 7.28	5.62 ± 12.44
Fold 2	87.80 ± 10.67	12.50 ± 40.14	87.03 ± 12.90	14.59 ± 40.91
Fold 3	87.86 ± 10.94	18.58 ± 54.84	87.41 ± 12.06	17.02 ± 53.09
Fold 4	88.81 ± 7.79	8.50 ± 23.82	89.04 ± 6.38	7.67 ± 23.36
Average	88.25 ± 10.19	10.57 ± 34.56	87.90 ± 10.94	10.77 ± 34.19
Median	91.39	0.98	90.97	0.99

DSC, Dice similarity coefficient; HD95, 95th percentile Hausdorff distance

### Networks

For the APE segmentation, this study employed two sophisticated neural network architectures: the widely recognized nnU-Net [17], which frequently achieves top rankings in segmentation challenge leaderboards, and the VT-UNet [18], a transformer-based model tailored for semantic segmentation tasks especially preserving local and global spatial relationships.

The nnU-Net utilizes heuristic methods and dataset-specific insights to automatically create three unique UNet [19] configurations, selecting the best-performing one via cross-validation. Its design builds upon a modified UNet framework, refined for optimal patch size selection and featuring instance normalization.

The VT-UNet [18] is a 3D volumetric transformer model developed specifically for medical image segmentation, inspired by the success of transformer-based approaches in computer vision. Its encoder divides the 3D input volume into smaller patches and processes them through linear embedding and merging layers in order to tokenize and combine adjacent spatial elements to build hierarchical features. Using window-based multi-head self-attention mechanisms, the encoder effectively captures both local and global contextual information. The decoder blends self-attention with cross-attention, integrating data from both encoder and decoder layers. This combination enhances the model’s capability to represent objects accurately, enabling precise pixel-level predictions of target regions.

**Fig. 2:**
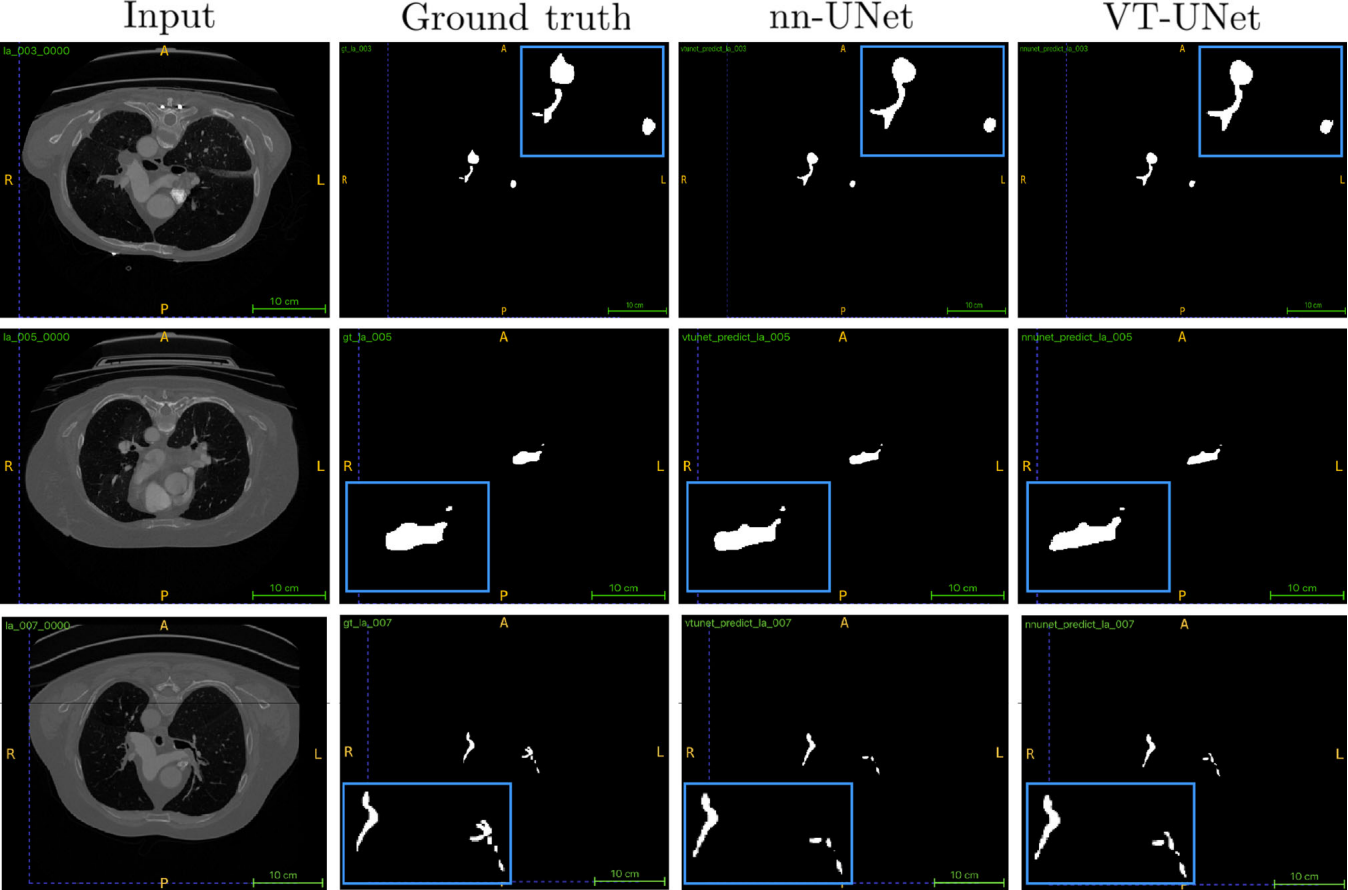
Example patient cases of the dataset. From left to right: input CT image, ground truth, nnU-Net predicted mask, VT-UNet predicted mask

### Experiments

The experiments were performed on a Linux-based server equipped with a 28-core Intel(R) Xeon(R) Gold 6330 CPU operating at 2.00 GHz. Additionally, an NVIDIA A100 GPU with 230 GB of memory was used to accelerate computational tasks. The software environment included Python 3.8 and PyTorch 2.2.

The nnU-Net was trained for 1,000 epochs using a learning rate of 0.01, a weight decay of 0.00003, and a combined Dice and cross-entropy loss function, optimized with the ADAM optimizer. Similarly, the VT-UNet was trained for 1,000 epochs with a fixed learning rate of 0.01, using stochastic gradient descent as the loss function and the ADAM optimizer. For both models, fivefold cross-validation was employed, and the reported final results were produced by averaging over all folds.

The dataset was randomly divided into 80% for training and 20% for validation, resulting in 160 training cases and 40 validation cases. The latter remained unseen during training per fold to ensure a robust evaluation of the models’ generalization capabilities.

## Results

The presented results list the average performance across all patient cases for each fold, followed by an aggregate average over all five cross-validation folds (see Table 1). The nnU-Net obtained a mean Dice similarity coefficient (DSC) of 88.25 ± 10.19 %, while the VT-UNet achieved 87.90 ± 10.94 %. The resulting 95th percentile Hausdorff distance (HD95) was on average 10.57 ± 34.56 mm and 10.77 ± 34.19 mm, respectively. Exemplary patient cases with their corresponding ground truth and networks’ predictions are illustrated in Fig. 2.

The training time per epoch for nnU-Net was approximately 27 s, whereas for VT-UNet, it was around 70 s. This disparity highlights the additional computational overhead associated with VT-UNet’s architectural complexity.

**Fig. 3:**
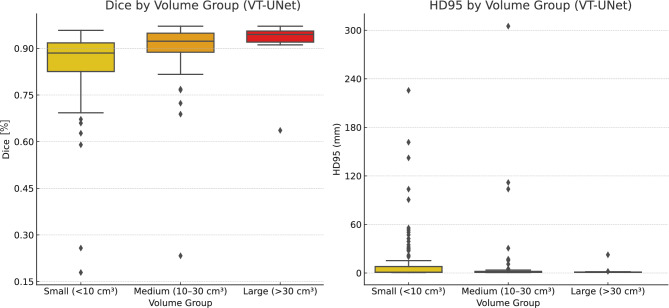
Dice scores and HD95 distances by volume group for the VT-UNet

**Fig. 4:**
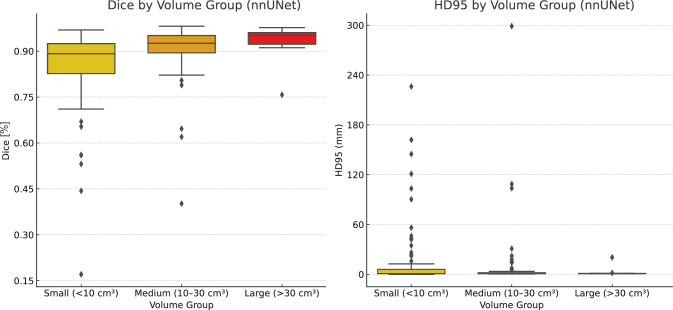
Dice scores and HD95 distances by volume group for the nnU-Net

The impact of the thrombus volumes on the segmentation accuracy was investigated by binning the data into three groups: small volumes with less than 10 cm^3^ (98 cases), medium volumes between 10 and 30 cm^3^ (90 cases), and large volumes greater than 30 cm^3^ (12 cases). This resulted in a clear trend, where the segmentation performance improved with increasing volume (see Figs. 3 and 4), even though large thrombi were significantly underrepresented. Both, nnU-Net and VT-UNet achieved the highest Dice scores and the lowest HD95 values for large thrombi, with mean Dice scores of 93.07 % and 91.70 %, and corresponding HD95 values of 2.57 mm and 2.80 mm, respectively. In contrast, small thrombi resulted in lower Dice scores with on average 85.55% for nnU-Net and 85.18 % for VT-UNet and larger HD95 distances with on average 13.70 mm and 14.30 mm, respectively. This highlights the difficulty of segmenting small thrombi due to limited spatial context. While both models perform similarly, VT-UNet produces slightly more compact distributions in the small volume group.

Outliers regarding the HD95 metric were most prevalent in the group with small thrombi. These cases indicated significant false positive boundary prediction errors, often caused by poorly defined or very small objects, as well as, for instance, by lung lesions, which were mistaken for APEs due to similar Hounsfield units and shape. For medium and large volumes, the results were more consistent, with tight interquartile ranges and fewer errors. Although both models produced outliers in general, VT-UNet showed fewer extreme values overall, suggesting slightly more stable boundary predictions in challenging cases.

**Fig. 5:**
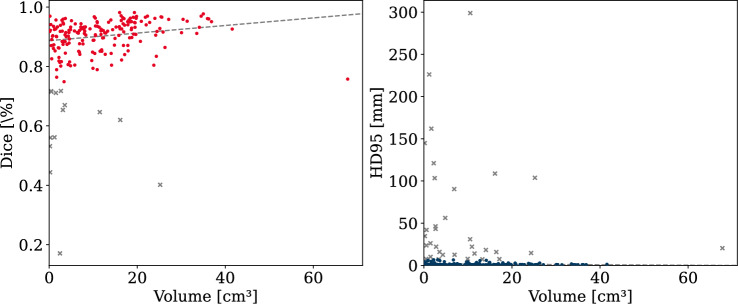
Correlation of Dice and HD95 with volume for nnU-Net (outliers excluded)

**Fig. 6:**
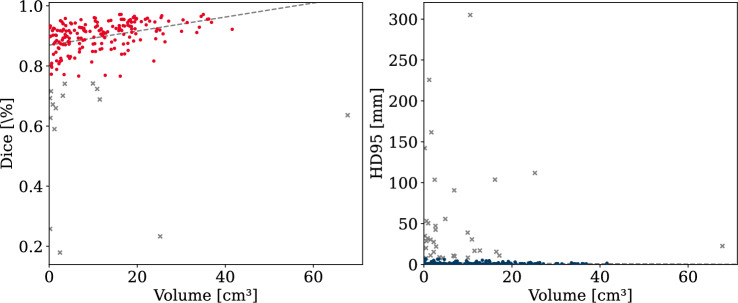
Correlation of Dice and HD95 with volume for VT-UNet (outliers excluded)

Figures 5 and 6 illustrate the relationship between thrombus volume and segmentation quality (Dice and HD95) after removing statistical outliers. For nnU-Net, the average Dice scores for small thrombi (with volume less than 10 cm^3^) increased from 85.55% to 89.95%, and the average HD95 improved from 13.70 to 1.55 mm after outlier removal. For large thrombi (greater than 30 cm^3^), the average Dice improved from 93.07% to 94.65%, and the average HD95 decreased from 2.57 to 0.95 mm. A similar trend was observed for VT-UNet, where small thrombi showed an increase in mean Dice from 85.18% to 89.67%, and a decrease in mean HD95 from 14.30 to 1.41 mm. For large thrombi, mean Dice improved from 91.70% to 94.25%, while the average HD95 dropped from 2.80 to 1.01 mm. These results highlight that the segmentation accuracy noticeably improved with increasing thrombus volume and that statistical outliers commonly occurred in the smaller volume group, hence the distinct improvement after their removal.

**Fig. 7:**
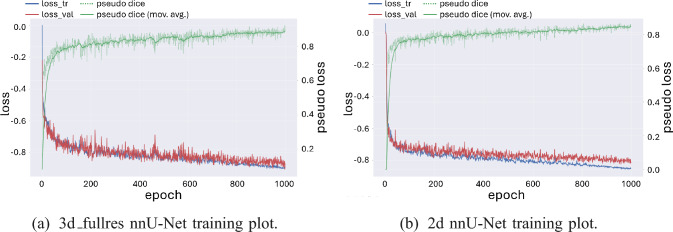
Training progress comparison between nnU-Net 2d and 3d_fullres configurations (fold 0). The plots show training and validation loss (left axis) along with pseudo Dice and its moving average (right axis)

Moreover, to assess the importance of volumetric information within the image input, we compared nnU-Net in its 2D and 3D_fullres modes across the first two folds. The 3D model consistently outperformed its 2D counterpart, achieving Dice scores of 87.73% and 89.04%, compared to 78.68% and 80.80%, respectively. Training curves (see Fig. 7) further highlight this difference: while both setups converged stably, the 3D configuration reached higher validation Dice values earlier and sustained them throughout training. This highlights the immense value of volumetric context in segmenting elongated or fragmented APE regions.

**Fig. 8:**
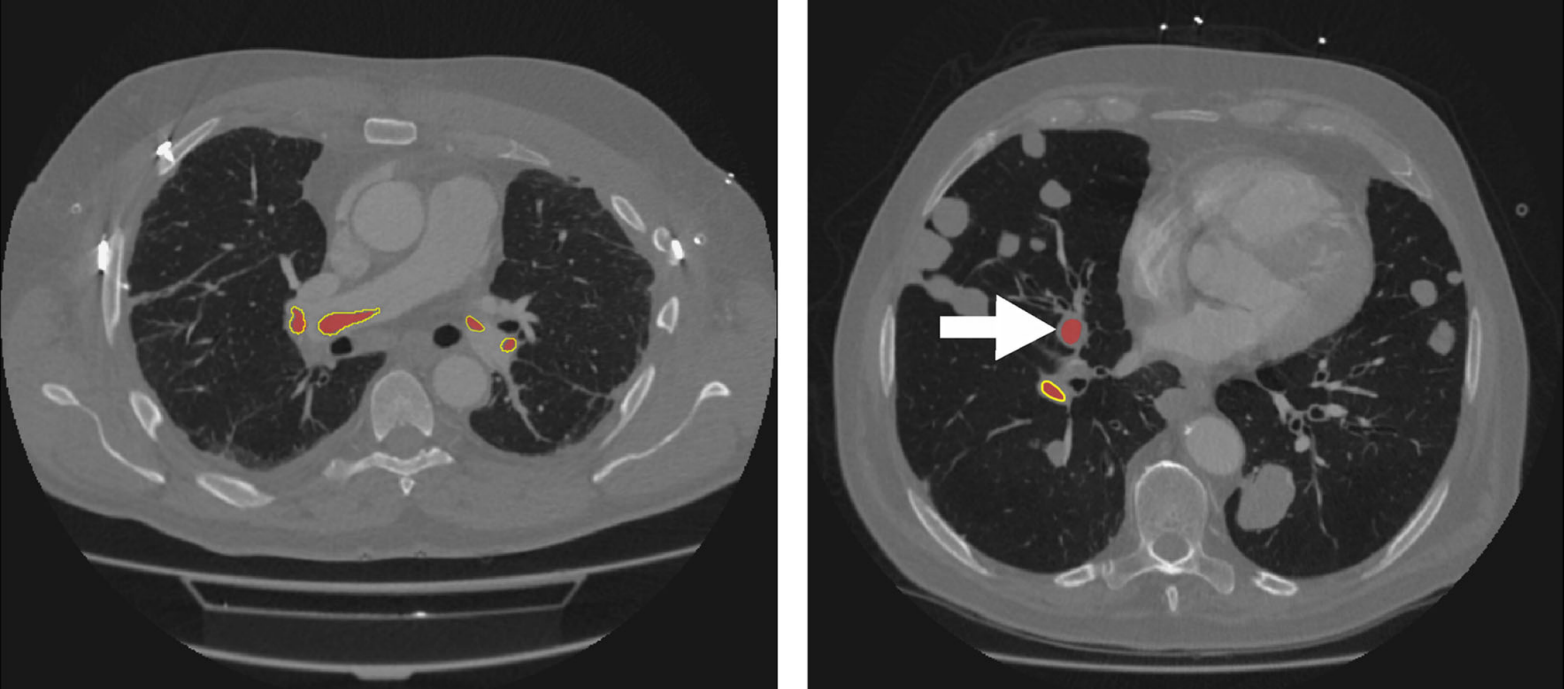
Exemplary patient cases and the corresponding VT-UNet’s results with high agreement to manual ground truth (left) and wrongly identified APE (right). For the latter, the VT-UNet predicts an APE in one of the metastases due to similar HU values and shape compared to the real APEs

## Discussion

The results of this study underscore the effectiveness of state-of-the-art neural network architectures like the nnU-Net and VT-UNet in addressing the challenges of APE segmentation in CTPA data. These architectures leverage innovative approaches to feature extraction and segmentation, significantly improving performance compared to traditional methods especially in comparison with native network variants of the UNet [16].

The nnU-Net’s robust performance, with an average DSC of 88.25 ± 10.19 % and mean HD95 of 10.57 ± 34.56 mm illustrates its adaptability and efficiency in capturing the complex spatial hierarchies of APE in CTPA images. In addition, it streamlines the procession pipeline due to automated hyperparameter tuning and validation schemes [17]. In comparison, the VT-UNet achieves a competitive average DSC score with 87.90 ± 10.94 %, while displaying a mean HD95 metric with 10.77 ± 34.19 mm. This comparatively large HD95 distances may be caused by false positive outliers, which could be observed in very few cases (see Fig. 8). Although it may not be of major concerns with respect to the clinical applicability, those outliers could be filtered out with false positive reduction strategies. Generally, both networks showed high agreement with the ground truth with hardly any APE parts missed out. Aside from above mentioned outliers, most discrepancies arose from smaller over- or undersegmentation at the rims of correctly identified APEs.

Most related works focused solely on categorical labeling [8, 9] and detection [10–13] of APE, while missing out on quantitative assessments of individual APEs, which however would have vast benefits in clinical treatment planning [3]. Currently, there are only limited works addressing the precise and automated segmentation of APEs in CT and CTPA images, respectively. Liu et al. [14] reported precision and *F*1-scores of $97.0\%$ and $96.7\%$ by applying their CAM-Wnet to an internal dataset of 2,700 2D CT slice images of 25 patient cases. In addition, four other networks variants, like the standard UNet or ResUNet were implemented and tested on the same dataset, although achieving inferior accuracies. While the reported segmentation accuracies indicate very high agreement with the ground truth, clinical applicability remains questionable due to the two-dimensional design of their approach with regard to the nature of common 3D CTPA imaging, as well as their focus on APEs located only in major pulmonary arteries. Pu et al. [15] applied their R2-UNet to the publicly available RSNA-PE dataset [20] as well as to a second internal test set. After training a network for APE candidate detection, volumetric patches were used for the segmentation step, achieving an average DSC of $67.6 \pm 16.8\%$. Therefore, the achieved segmentation accuracy of Pu et al. [15] is significantly lower, while being computed on smaller volumetric patches, compared to the presented work with $88.25\%$ DSC achieved on whole 3D CTPA images. More recently, Chen et al. [16] proposed a Swin Unet variant called SCUNet++ and applied it to two datasets consisting of 8,792 2D CTPA images of 35 patients and 8,900 2D CTPA images of 91 scans, respectively. The first mentioned dataset originated from the publicly available FUMPE dataset [21], while being re-annotated in parts by collaborating clinicians, which may hamper comparability. The achieved segmentation accuracy was on average $83.5\,\%$ on the edited FUMPE dataset and $83.4\,\%$ on the second internal dataset. Similar to Liu et al. [14], the network’s input were 2D CTPA images. Hence, the reported accuracies are inferior compared to our results, which were computed on whole 3D CTPA image volumes. However, such comparisons are merely of an indirect manner, due to different data bases. In general, our experimental results indicate current state-of-the-art performance, especially regarding whole-scan volumetric CTPA imaging (Table 2).

**Table 2:** Comparison of relevant related works with the nnU-Net and VT-UNet adapted to our dataset

Work	Image input	DSC (%)	HD95 (mm)
Liu et al. [14]	2D CT slices	96.6	–
Pu et al. [15]	3D CT patches	67.6 ± 16.8	–
Chen et al. [16]	2D CT slices	83.47 ± 6.12	3.83 ± 1.02
nnU-Net	Whole 3D CT scans	88.25 ± 10.19	10.57 ± 34.56
VT-UNet	Whole 3D CT scans	87.90 ± 10.94	10.77 ± 34.19

DSC, Dice similarity coefficient; HD95, 95th percentile Hausdorff distance

Limitations regarding the presented work comprise the inter-reader variability since the manual segmentations have only been supervised by two experienced radiologists. Including more readers as well as multi-center datasets would further promote the models generaliziability and robustness. In addition, pathologic cases, e.g., presented in Fig. 8 suffers from multiple lung metastases, may hamper the training of the network and a possible preprocessing step could account for a classification of disease state first and applying the training of a CNN afterward.

The segmentation of acute pulmonary embolism in CT pulmonary angiograms has the potential to be a crucial factor for patient-individual risk stratification. Linking derived parameters like thrombus volume and extent with additional biomarkers like vascular occlusion and regional perfusion impairment could promote statements regarding right ventricular dysfunction and short-term mortality [3]. By combining APE segmentation with anatomical references (e.g., from the TotalSegmentator [22]), our approach aims to automatically quantify occlusion extent in specific vascular territories. To evaluate the clinical utility of such a framework, prospective studies in collaboration with our clinical partners at the University Hospital of Schleswig-Holstein are planned. These studies will assess whether automated extraction of such parameters improves diagnostic accuracy, accelerates treatment decisions, shorten confirmation time, and contributes to better clinical outcomes in patients with APE.

## Conclusion

In this study, we investigated the performance of nnU-Net and VT-UNet for the segmentation of acute pulmonary embolism in CT pulmonary angiograms. Both architectures demonstrated superior performance compared to related works, with nnU-Net achieving an average DSC of 88.25 % and a HD95 of 10.57 mm. VT-UNet produced DSC scores with on average 87.90 %, while resulting in a HD95 distance of 10.77 mm. The findings affirm the potential of these state-of-the-art models to address critical challenges in APE diagnosis and treatment planning, offering improvements in overall segmentation accuracy and reliability. Future research could explore further optimization of these architectures, incorporation of multi-modal data, and their real-world application in clinical settings.

## Data Availability

The trained model developed in this study is publicly available at: https://github.com/AIinMedicalApplications/APES_ThrombusSegmenter.
